# *Staphylococcus aureus* Nasal Colonization among Children with Sickle Cell Disease at the Children’s Hospital, Accra: Prevalence, Risk Factors, and Antibiotic Resistance

**DOI:** 10.3390/pathogens9050329

**Published:** 2020-04-28

**Authors:** Vera A. Appiah, George A. Pesewu, Fleischer C. N. Kotey, Alahaman Nana Boakye, Samuel Duodu, Edem M. A. Tette, Mame Y. Nyarko, Eric S. Donkor

**Affiliations:** 1 Department of Medical Laboratory Science, School of Biomedical and Allied Health Sciences, University of Ghana, Legon P. O. Box LG 54, Accra, Ghana; 2 FleRhoLife Research Consult, Teshie P. O. Box TS 853, Accra, Ghana; 3 Department of Medical Microbiology, University of Ghana Medical School, Accra P. O. Box 4236, Ghana; 4 West African Centre for Cell Biology of Infectious Pathogens, University of Ghana, Legon P. O. Box LG 54, Accra, Ghana; 5 Department of Biochemistry, Cell and Molecular Biology, University of Ghana, Legon P. O. Box LG 54, Accra, Ghana; 6 Department of Community Health, University of Ghana Medical School, Accra P. O. Box 4236, Ghana; 7 Princess Marie Louise Children’s Hospital, Accra P. O. Box GP 122, Ghana

**Keywords:** *S. aureus*, MRSA, colonization, antibiotics, Accra

## Abstract

The aim of this study was to investigate *S. aureus* carriage among children with sickle cell disease (SCD), including the prevalence, risk factors, and antibiotic resistance. The study was cross-sectional, and involved 120 children with SCD recruited at the Princess Marie Louise Children’s Hospital (PML) in Accra and 100 apparently healthy children from environs of the hospital. Nasal swab samples were collected from the study participants and cultured for bacteria. Confirmation of *S. aureus* and methicillin-resistant *Staphylococcus aureus* (MRSA) isolates were done using the tube coagulase test and *mec*A polymerase chain reaction, respectively. All the *S. aureus* isolates were tested against standard antimicrobial agents using the Kirby-Bauer method. A structured questionnaire was used to obtain the socio-demographic and clinical data of the study participants. Binary logistic regression was used to identify determinants of *S. aureus* and MRSA carriage among the study participants. The nasal carriage prevalence of *S. aureus* was 33.3% (*n* = 40) and 10% (*n* = 10) among the participants of the SCD and control groups, respectively. As regards MRSA nasal carriage prevalence, the respective values were 3.33% (*n* = 4) and 0.00% (*n* = 0). SCD was significantly associated with *S. aureus* colonization (*p* < 0.0001, OR = 4.045), but not MRSA colonization (*p* = 0.128). In the SCD group, the significant predictors of *S. aureus* carriage were increasing age (*p* = 0.003; OR = 1.275) and living in self-contained apartments (*p* = 0.033; OR = 3.632), whereas male gender (*p* = 0.018; OR = 0.344) and the practice of self-medication (*p* = 0.039; OR = 0.233) were protective of *S. aureus* carriage. In the control group, a history of hospitalization in the past year was a risk factor for the carriage of *S. aureus* (*p* = 0.048; OR = 14.333). Among the participants of the SCD and control groups, respectively, the resistance prevalence recorded by *S. aureus* against the various antibiotics investigated were penicillin (100% each), cotrimoxazole (27.5% vs. 20%), tetracycline (25% vs. 50%), rifampicin (82.5% vs. 50%), erythromycin (30% vs. 20%), clindamycin (32.5% vs. 50%), gentamicin (7.5% vs. 20%), cefoxitin (27.5% vs. 20%), linezolid (30% vs. 40%), and fusidic acid (95% vs. 80%). The proportion of *S. aureus* isolates that were multidrug resistant (MDR) was 92.5% (37/40) in the SCD group and 100% (10/10) in the control group.

## 1. Introduction

*Staphylococcus aureus* is one of the predominantly isolated Gram-positive bacteria in clinical specimens, causing infections such as pneumonia, septicaemia, endocarditis, osteomyelitis, and meningitis [1,2,3,4]. It is also a commensal of various parts of the body, including the skin, perineum and pharynx, but preferentially colonizes the moist squamous epithelium of the anterior nares of the nose [5,6]. Consequently, different categories of *S. aureus* carriers—persistent carriers, intermittent carriers, and non-carriers—have been reported at rates 20%, 30%, and 50%, respectively [5,7,8,9].

The clinical significance of *S. aureus* has been accentuated by the emergence and spread of methicillin-resistant strains (methicillin-resistant *Staphylococcus aureus* [MRSA]), which are characteristically different from methicillin-sensitive strains (methicillin-sensitive *Staphylococcus aureus* [MSSA]) by virtue of their resistance to all beta-lactam antibiotics. MRSA strains were previously nosocomial, and bore the tag healthcare-associated MRSA (HA-MRSA) [10], but now bear additional tags—community-associated MRSA (CA-MRSA) and livestock-associated MRSA (LA-MRSA)—owing to their association with communities and livestock, respectively [11,12,13,14]. Over the years, the proportion of isolation of MRSA among *S. aureus* infections has increased to as high as 70% [15,16,17,18,19]. In the United States, in the year 2014, it accounted for an estimated 72,444 invasive infections, resulting in 9194 deaths [20]. Inpatient stays due to MRSA infections cost USD 14,000, relative to the financial burden for all other stays (USD 7600), with a two-fold increase in the length of hospitalization [21,22].

Sickle cell disease (SCD) refers to a couple of genetic disorders that are associated with structurally abnormal haemoglobin. The main genotypes that give rise to SCD include Hb SS, Hb SC, Hb Sβ^+^-thalassemia, and Hb Sβ^0^-thalassemia; other rare forms include haemoglobin SD and haemoglobin SE [23]. Owing to the impairment of their immune system, SCD patients are relatively predisposed to bacterial infections, resulting in frequent hospitalization and the intake of antibiotic treatment. This could invariably select for antibiotic resistant pathogens among these individuals, and potentially make them reservoirs for multidrug-resistant commensal and pathogenic microbes, such as MRSA. Indeed, MRSA carriage is an antecedent to subsequent infections [6,24,25]. Since 2012, MRSA has caused several outbreaks in Ghana [26], and a replication of these outbreaks in SCD patients could be fatal. Notwithstanding the public health threat that MRSA could pose to SCD patients, this immunologically-challenged population has received little attention from researchers studying MRSA carriage. Microbiology researchers studying sickle cell disease have usually focused on the occurrence of *Streptococcus pneumoniae* in SCD patients [27,28]. Yet, among SCD patients, *S. aureus* holds more potential in causing invasive diseases than *S. pneumoniae* [29,30,31,32,33]. Moreover, MRSA carriage studies seem to have focused on the general population [34,35,36] and a few other risk groups, such as children [37] and persons with HIV infection [38,39,40]. Hence, knowledge on the epidemiology of MRSA, including carriage rates, determinants of carriage, and the antibiotic resistance of colonizing strains, in relation to SCD patients, is limited. Therefore, conducting MRSA surveillance studies among these individuals would provide additional insights about the epidemiology of the pathogen and data to guide the management of its infections in this at-risk population. On this premise, this study aimed at investigating *S. aureus* and MRSA colonization among children with SCD at a paediatric hospital in Accra, Ghana, focusing on the prevalence, risk factors, and antimicrobial resistance patterns.

## 2. Methodology

### 2.1. Study Site, Design, and Sampling

This study was conducted at the Princess Marie Louise Children’s Hospital (PML) in Accra, Ghana. The city hosts about two million people, and has eight government or quasi-governmental hospitals (http://www.statsghana.gov.gh/). Apart from the Korle Bu Teaching Hospital, which offers specialized paediatric care as one of its specialties, PML is the only other major public hospital in the Accra Metropolis that focuses primarily on pediatric care. Its sickle cell clinic attends to SCD patients once a week on Thursdays. On average, about 30 children attend the clinic weekly. The study was cross-sectional, and involved 120 children with SCD (both HbSS and HbSC genotypes) and 100 children without the disease (control group) recruited between March and August 2018. The SCD participants were recruited from the sickle cell clinic of the hospital, and the control group participants were recruited from the environs of the hospital. The inclusion criteria for the selection of the participants constituting the SCD group were: being in a steady state with laboratory-proven HbSS or the HbSC genotype, being between 1 and 13 years of age, and being an outpatient. In the selection of the participants of the control group, the inclusion criteria satisfied were: being a child with laboratory-proven HbAA genotype, being apparently healthy, and being between 1 and 13 years of age. In both study groups, the exclusion criteria were: being on antimicrobials (other than penicillin, since in Ghana, penicillin is routinely administered to SCD individuals as prophylaxis) two weeks prior to sampling, having known co-morbidities, and an inability to determine the haemoglobin genotype. With the aid of a structured questionnaire, data on potential risk factors for *S. aureus* and MRSA carriage were collected from the study participants.

Furthermore, swabs were obtained from the anterior nares of the study participants by a qualified paediatrician; this was preceded by obtaining informed consent from their parents/guardians. For every participant, a sterile cotton swab was rotated five times in both anterior nares. Each sample was thereafter secured in a pre-labeled vial containing 1ml skim milk-tryptone-glucose-glycerin (STGG) medium. Subsequently, the samples were sent to the research laboratory of the Department of Medical Microbiology, University of Ghana Medical School, to be processed within four hours after collection. Upon arrival, these samples were each vortexed for about two minutes, followed by refrigeration at −80 °C, until needed.

### 2.2. Laboratory Analysis

The processing of the specimens, identification of *S. aureus* and MRSA, antimicrobial susceptibility testing, and molecular analysis were done following the procedures described by Donkor et al. [40]. The specimens were cultured on chocolate agar, blood agar, and MacConkey agar, and presumptive staphylococcal isolates were identified based on their reaction to the tube coagulase test. All isolates whose responses were negative were identified as coagulase-negative staphylococci (CoNS). Those isolates that displayed positive results to the test were presumptively identified as *S. aureus*, and were subjected to antimicrobial susceptibility testing. The testing was performed and interpreted following guidelines of the Clinical and Laboratory Standards Institute (www.clsi.org) using the following antimicrobials: linezolid (10 µg), cefoxitin (30 µg), fusidic acid (10 µg), clindamycin (2 µg), penicillin (10 µg), cotrimoxazole (1.25 µg trimethoprim + 23.75 µg sulphamethoxazole), rifampicin (5 µg), gentamicin (10 µg), erythromycin (15 µg), and tetracycline (30 µg). Subsequently, the cefoxitin-resistant isolates were confirmed as *S. aureus* via polymerase chain reaction (PCR) targeting the *nuc*A gene and MRSA via PCR targeting the *mec*A gene.

### 2.3. Data Analysis

The data were analyzed with the help of STATA 14 (Strata Corp, College Station, TX, USA). Data on the resistance of *S. aureus* to the antimicrobials tested were summarized using descriptive statistics. Fisher’s exact tests were performed to determine the association between SCD and *S. aureus* and MRSA colonization. Within each group, independent samples Chi-square tests were performed to determine associations between individual categorical risk factors and *S. aureus* and MRSA colonization. Point biserial correlations were also performed to determine associations between risk factors that were continuous variables and *S. aureus* and MRSA colonization. Finally, risk factors that showed significant associations with colonization in the Chi-square and point biserial correlation tests were put in a binary logistic regression model to determine their predictive value of colonization. The significance of each predictor variable of colonization was assessed by determining the *p* value, odds ratio, and confidence interval; *p* values less than 0.05 were considered significant.

### 2.4. Ethical Approval

Approval for the conduction of this study was given by the Ethical and Protocol Review Committee of the School of Biomedical and Allied Health Sciences, College of Health Sciences, University of Ghana, with protocol identification number “SBAHS—MD.//AA/5A/2016-2017”.

## 3. Results

### 3.1. Demographic, Household, and Clinical Features of the Study Participants

In total, two hundred and twenty (220) individuals participated in this study. Of this number, the SCD participants comprised one hundred and twenty (120), whereas the participants of the control group were one hundred (100). The mean age and BMI of the SCD participants were 5.84 years and 15.37 Kg/m^2^, respectively, and the corresponding values within the control group were 6.36 years and 19.80 Kg/m^2^ respectively. In the SCD group, the gender distribution was uneven (48.3% males vs. 51.7% females), but was even in the control group (the males and females comprised 50% each of the population). In both study groups, the majority of the participants were enrolled in school (86.7% in the SCD group vs. 97% in the control group), resided in compound houses (69.2% in the SCD group vs. 61% in the control group), and often washed their hands with soap (58.3% in the SCD group vs. 55.0% in the control group), and at least 97% of the participants had no health worker present in their household. Table 1 presents the demographic and household characteristics of the study participants.

The clinical features of the study participants are presented in Table 2. As evident in the table, the SCD participants were predominantly (96.7%) on penicillin prophylaxis, whereas that attribute was absent among the participants of the control group. Moreover, relatively few of the study participants reported that they practiced self-medication (20% in the SCD group vs. 23% in the control group), had a history of hospitalization in the past year (40.8% in the SCD group vs. 8.0% in the control group), or a history of pneumonia (6.7% in the SCD group vs. 0.0% in the control group).

### 3.2. Relationship between Sickle Cell Disease and Staphylococcal Carriage

The distribution of Staphylococci isolated from the study participants—SCD group relative to the control group—were as follows: *Staphylococcus aureus* (33.3% vs. 10%), MRSA (3.33% vs. 0.00%), and coagulase-negative staphylococci (7.5% vs. 8.0%). Considering both study groups as a composite, the overall nasal carriage prevalence of *S. aureus*, MRSA, and coagulase-negative staphylococci were 22.73% (*n* = 50), 1.82% (*n* = 4), and 7.73% (*n* = 17) respectively. Moreover, a significant association was observed between the presence of sickle cell disease and *S. aureus* carriage (*p* < 0.0001), but this was not observed in relation to MRSA carriage (*p* = 0.128). The odds ratio for *S. aureus* carriage in relation to the presence of SCD was 4.045. This means that SCD children have more than a four-fold increased risk for *S. aureus* carriage.

PCR targeting the *mec*A gene did not yield evidence supporting the presence of the *mec*A gene in twelve (75%) of the sixteen cefoxitin-resistant isolates. It is noted that all these isolates are *nuc*A gene-confirmed *S. aureus* isolates.

### 3.3. Risk Factors for Colonization with S. aureus and MRSA among the Study Participants

The results of the logistic regression analysis indicated that among the SCD children, male gender and the practice of self-medication were protective of *S. aureus* carriage, whereas increasing age, and living in self-contained apartments predisposed individuals to *S. aureus* carriage. In the control group, a history of hospitalization in the past year was a risk factor for *S. aureus* carriage. No risk factors of MRSA carriage were identified in either of the study groups. Details of the risk factor analysis are presented in Table 3.

### 3.4. Patterns of Antimicrobial Resistance among the S. aureus and MRSA Isolates

**Figure 1:**
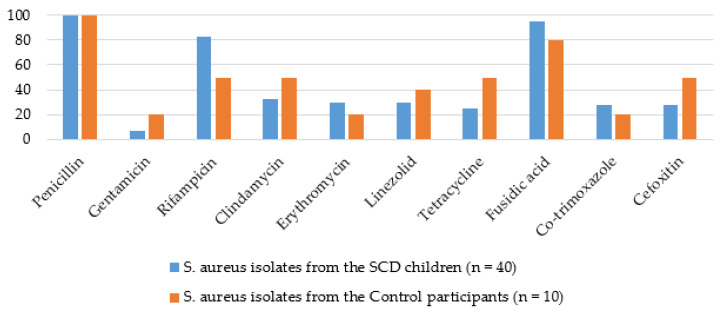
Antibiotic resistance prevalence of the *S. aureus* isolates.

In both study groups, all the *S. aureus* isolates were penicillin-resistant. Moreover, relatively higher rates of resistance were observed for fusidic acid (95% in the SCD group vs. 80% in the control group) as well as rifampicin (82.5% in the SCD group vs. 50% in the control group), which was the only antibiotic whose differences in resistance rates reached statistical significance (z = 2.152; *p* = 0.03). The rates recorded against the other antimicrobials were: gentamicin (7.5% in the SCD group vs. 20% in the control group), clindamycin (32.5% in the SCD group vs. 50% in the control group), erythromycin (30% in the SCD group vs. 20% in the control group), linezolid (30% in the SCD group vs. 40% in the control group), tetracycline (25% in the SCD group vs. 50% in the control group), co-trimoxazole (27.5% in the SCD group vs. 20% in the control group), and cefoxitin (27.5% in the SCD group vs. 50% in the control group). The rates of multidrug resistance (resistance to more than two antimicrobial classes [41]) were 92.5% in the SCD group (*n* = 37) and 100% (*n* = 10) in the control group. In Figure 1, the prevalence rates of antimicrobial resistance are presented.

## 4. Discussion

This study aimed to investigate *S. aureus* and MRSA colonization among children with sickle cell disease and those without the disease in Accra. As far as we know, it is the second study on *S. aureus* and MRSA colonization among SCD children in Ghana, and one of the limited MRSA carriage studies conducted among this risk group globally. Overall, among the participants recruited in this study, the presence of SCD predisposed to the nasal carriage of *S. aureus* at a higher rate (33.3% vs. 10%). In a previous study that partly focused on comparing cohorts of children with and without SCD in the country on their predisposition to *S. aureus* carriage [42], the *S. aureus* carriage prevalence was not significantly different. In fact, the carriage prevalence was higher in the control group (50%) than the SCD group (48%). Another contrasting observation to that of this study is seen in one similar study conducted in Gabon [43], which reported a finding similar to that of Donkor et al. [42]—46.6% of the SCD children were *S. aureus* carriers as opposed to 46.9% in the control group. Consequently, this study is apparently the first to identify people with SCD as a risk group for *S. aureus* carriage, which could be due to changes in the epidemiology of prophylactic measures for SCD patients against *S. pneumoniae*, such as penicillin use and vaccination, which are known to affect *S. aureus* colonization [42]. Although additional studies could be conducted to ascertain this finding, this observation partly explains the high occurrence of invasive diseases caused by *S. aureus* in SCD patients [29,30,31,32,33]. The identification of individuals with SCD as an at-risk population for *S. aureus* carriage is an additional reason for worry, given their high intake of antibiotics, as this could make them reservoirs of multidrug-resistant *S. aureus*. Interestingly, in this study, this risk group for *S. aureus* carriage was not predisposed to MRSA carriage—the prevalence was 3.33% in the SCD group as opposed to 0.00% in the control group. It is noted that this is not the first time that *S. aureus* carriage, but not MRSA carriage, has been associated with a risk group. A previous study in the region identified HIV-infected individuals as a risk group for *S. aureus* carriage, but not MRSA carriage [40]. In a previous report by Schaumburg et al. [44], immunosuppression was noted to be associated with the carriage of PVL-positive *S. aureus*. As PVL characterization was not carried out in the current study and that of Donkor et al. [40], an exhaustive inference cannot be drawn between the PVL status of colonizing *S. aureus* strains and immunosuppression. However, as both studies were carried out in a PVL-endemic region, it is reasonable to hypothesize that PVL carriage could be a possible explanation for the linkage between immunosuppression and *S. aureus* carriage in the current study and the study conducted by Donkor et al. [40]. These observations reinforce the need for the continued surveillance of *S. aureus* and MRSA among at-risk populations.

In our study, among the SCD participants, none of the factors studied were predictive of MRSA carriage. However, male gender and the practice of self-medication were protective of *S. aureus* carriage, while increasing age and living in self-contained apartments were identified as risk factors for *S. aureus* nasal carriage. In the control group, a history of hospitalization in the past year was a risk factor for *S. aureus* nasal carriage. Some studies have failed to demonstrate any significant association between gender and *S. aureus* carriage [34,45,46]. Be that as it may, male gender has been associated with *S. aureus* carriage in other studies [24]. Consistent with these reports, female gender, by virtue of estrogen production, has been linked with better immune function [47]. It is just a few earlier studies that reported female gender as a risk factor for *S. aureus* carriage [48]. Hence, the reason why male gender emerged to be protective of *S. aureus* carriage among the SCD participants in the current study is unclear. It is possible that the female participants in this study engaged more frequently in nose picking, a phenomenon that is known to predispose to *S. aureus* carriage [6]. At present, this only remains a hypothesis, as the frequency of nose picking was not evaluated in this study. Another possibility is that the nasal secretions of the male participants in this study were more bactericidal. It has been reported that nasal fluids from non-carriers are more bactericidal to *S. aureus* than are those from carriers [49]. Also likely, the SCD participants’ serum levels of 25-hydroxyvitamin D, a marker of total levels of vitamins in the body, played a role. An inverse relationship has been reported between 25-hydroxyvitamin D and *S. aureus* carriage [50], and SCD patients are known to be frequently vitamin D-deficient [51]. Perhaps, the male SCD participants in the current study had significantly higher levels of this marker than did their female counterparts, and this likely accounted for their low levels of *S. aureus* carriage. Some of these hypotheses may be additional dynamics that the presence of SCD introduces in terms of gender-contingent *S. aureus* carriage. Additional research could help improve clarity on this atypical finding.

It is quite interesting that the practice of self-medication emerged to be protective of *S. aureus* colonization among the SCD children. This is especially so, given that a previous study by Lemma et al. [52] reported a history of antibiotic use over the previous 3 months to be a significant risk factor for *S. aureus* colonization among children aged between 7 and 15 years. However, the researchers failed to indicate whether those individuals with a history of antibiotic usage administered the drugs by themselves (self-medication) or with doctors’ prescription. Hence, it does not seem legitimate to compare the findings of this current study with that reported by Lemma et al. [52]. Moreover, even though antibiotic use has been reported to predispose to MRSA carriage [53], it is noted that the current study evaluated whether or not the study participants practiced self-medication, but did not evaluate the drugs used in the practice. Moreover, the variable “self-medication” is limited by the fact that it only encompasses what was reported by the study participants, and hence may not reflect self-medication in the absolute sense. Hence, a measure of caution is warranted in the interpretation of this finding. Regardless of the aforementioned technicalities, this finding underpins the need to evaluate self-medication practices in an exhaustive manner when carrying out *S. aureus* and MRSA surveillance studies, as well as the need to focus more studies on self-medication.

Furthermore, age has been noted to have an inverse relationship with *S. aureus* carriage [54,55,56,57,58]. For example, carriage rates of 28.4% and 35% have been reported in children aged from four to six years [56] and three to eleven years, respectively [57]. In a more recent study, Halablab et al. [58] reported carriage rates of 57.1%, 34.9%, 24.8%, 37.0%, and 45.8%, respectively, for individuals aged between 6–10 years, 11–17 years, 18–25 years, 26–40 years, and 41–65 years. In summary, they reported carriage rates of 43.9% among individuals aged between six and seventeen years as opposed to 29.3% among those aged between eighteen and sixty-five years). Hence, the contrasting finding that increasing age is a risk factor for *S. aureus* carriage among individuals with SCD is difficult to explain. It may be a chance finding, or could probably be attributed to differences in population dynamics, and there may be a need to rely on future research to improve insights on the observation.

Similar to the phenomenon with age in the current study, it is difficult to pinpoint why living in self-contained apartments would predispose to *S. aureus* carriage, as was observed among the SCD participants in this study. One possible explanation for the phenomenon could be that the *S. aureus* carriers among the SCD participants may have acquired their carrier states from colonized household members. Past studies have demonstrated the importance of close contacts within households and with parents in the spread of *S. aureus* carriage among children [59,60]. Nonetheless, as this study was not designed to include an evaluation of *S. aureus* carriage among household members of the study participants, these attempts at explanations are at best, educated guesses.

Finally, the observed predisposition of individuals with a history of hospitalization in the control group to *S. aureus* nasal carriage was not surprising. Yet, it is important to highlight that it has not been universally established as a risk factor. To illustrate, although some studies have failed at demonstrating previous hospitalization as a risk factor for *S. aureus* carriage [24,52], others have demonstrated that it predisposes to MRSA carriage [23,61,62]. Thus, this study seems to be the first to report a history of hospitalization as a risk factor for *S. aureus* colonization. It provides additional insights into the predisposition of individuals whose household members are employed in the healthcare setting to *S. aureus* carriage [53,63].

In both study groups, all the *S. aureus* isolates were penicillin-resistant, and the majority of them, at least, 80%, displayed resistance towards fusidic acid. The high rates of resistance recorded against penicillin was expected, given the high rates of resistance (>80%) recorded against the antibiotic by *S. aureus* in several studies [34,35,38,42,64]. These studies additionally reported *S. aureus* resistance to fusidic acid at rates of 0%–12%, and the high rates of fusidic acid resistance observed in this study could either be an isolated case or could predict the beginning of rendering fusidic acid ineffective as a therapeutic agent against *S. aureus* infections. For erythromycin, linezolid, and co-trimoxazole, the rates of resistance against them were similar in both groups, and generally ranged between 20% and 40%. Earlier studies conducted within the country [34,35,38,42,64] have reported comparable rates for erythromycin, but higher rates for co-trimoxazole. With regard to linezolid resistance, although the rates of 30% and 20% reported in the SCD and control groups, respectively, might seem moderate in this study, they are cause for worry. This is because generally, *S. aureus* resistance to linezolid is rare [65,66,67,68], and the situation is no different in Ghana [34,40]. Moreover, the antibiotic is one of the few therapeutic options for treating MRSA infections. Besides, it has a low coverage in the country. Evidently then, it may be necessary to design studies to screen for the plasmid-borne *cfr* gene as well as other linezolid resistance determinants among pathogens in the country. Although there seemed to be disparities between the two study groups with regard to the rates of resistance of the *S. aureus* isolates to gentamicin, clindamycin, and tetracycline, the apparent disparities may constitute a chance finding. Nonetheless, the rates of resistance are consistent with what have been reported in previous studies [39,40,64]. Resistance to rifampicin was significantly higher in the isolates emanating from the SCD group than those of the control group. This high rate (82.5%) warrants attention, particularly owing to the drawback it could pose to tuberculosis (TB) management should this resistance trait be transferred to etiologic agents of TB, given that rifampicin is a backbone to TB management [69].

It is noted that the phenotypic testing (cefoxitin resistance trait) upon which methicillin resistance was presumptively evaluated yielded results that were inconsistent with the *mec*A PCR results. Only four of the total sixteen cefoxitin-resistant *S. aureus* isolates, all emanating from the SCD participants, were confirmed to carry the *mec*A gene. Those isolates in which carriage of the *mec*A gene could not be demonstrated likely harbored other determinants of methicillin resistance, such as the newly discovered *mec*C gene [70,71].

The rates of multidrug resistance were high in both study groups (SCD group (92.5%, *n* = 37); control group (100%, *n* = 10)). These rates are higher than those recorded in previous studies [35,39,40,64], and may be due to the extensive marketing of antimicrobials and indiscriminate antibiotic use [54,55,72,73]. Evidently, policy makers and implementers would have to step up public health campaigns against antimicrobial abuse and institute more stringent policies on antimicrobial acquisition.

There are a few potential limitations to our study. Firstly, we sampled only the anterior nares (the main site of *S. aureus* colonization) and, therefore, our data does not cover other colonization sites, such as the skin, throat, and rectum. Secondly, the PCR analysis did not cover the newly discovered *mec*C gene.

## 5. Conclusions

This study concludes that among children in Accra, sickle cell disease predisposed to the carriage of *S. aureus*, but not MRSA. The risk factors for *S. aureus* colonization appeared to be quite different in the SCD children compared with the control group. Both the SCD children and the control group harbored multidrug-resistant *S. aureus*, and this may be due to extensive antimicrobial use in the country. Further research involving longitudinal studies are needed to provide insights into the dynamics of *S. aureus* and MRSA carriage among SCD patients in Ghana. Such studies could also be extended to other at-risk populations that have not been previously studied, such as stroke patients [74].

## Figures and Tables

**Table 1:** Demographic and household characteristics of the study participants.

Demographic and Household Characteristics	SCD Group		Control Group	
	Number	%	Number	%
**Gender**				
Male	58	48.3	50	50.0
Female	62	51.7	50	50.0
**Current school enrolment**				
Yes	104	86.7	97	97.0
No	16	13.3	3	3.0
**Type of residence**				
Self-contained	37	30.8	38	38.0
Compound	83	69.2	61	61.0
**Presence of health worker in household**				
Yes	3	2.5	0	0
No	117	97.5	100	100
**Hand washing with soap**				
Rarely	50	41.7	45	45.0
Often	70	58.3	55	55.0

Age (SCD group = 5.84 ± 2.99 years; Control group = 6.36 ± 3.45); BMI (SCD group = 15.37 ± 10.91 Kg/m^2^; Control group = 19.80 ± 9.45 Kg/m^2^); Number of individuals per household (SCD group = 8.14 ± 3.32; Control group = 7.14 ± 2.80).

**Table 2:** Clinical features of the study participants.

Clinical Features	SCD Group		Control Group	
	Number	%	Number	%
Self-reported self-medication *				
Yes	24	20	23	23.0
No	96	80	77	77.0
**Penicillin prophylaxis**				
Yes	116	96.7	0	0
No	4	3.3	100	100
**History of hospitalization in the past year**				
Yes	49	40.8	8	8.0
No	71	59.2	92	92.0
**Chronic skin condition**				
Yes	0	0	0	0
No	120	100	100	100
**History of pneumonia**				
Yes	8	6.7	0	0
No	112	93.3	100	100
**History of TB**				
Yes	0	0	0	0
No	120	100	100	100
**History of surgery**				
Yes	0	0	0	0
No	120	100	100	100
**Underlying disease**				
Yes	0	0	0	0
No	120	100	100	100
**History of blood transfusion**				
Yes	42	35	0	0
No	78	65	100	100

Frequency of hospitalization in the past year (SCD group = 0.70 ± 1.37 times; Control group = 0.10 ± 0.41); * Refers to participants’ use of medications without doctors’ prescription.

**Table 3:** Risk factors for *S. aureus* colonization.

	SCD Group		Control Group	
Risk Factor	OR (95% CI)	*p* Value	OR (95% CI)	*p* Value
**Age**	1.275 (1.084–1.499)	0.003	N/A	N/A
**Male gender**	0.344 (0.142–0.833)	0.018	N/A	N/A
**Living in SC apartments**	3.632 (1.108–11.906)	0.033	N/A	N/A
**Practice of self-medication**	0.233 (0.059–0.927)	0.039	N/A	N/A
**Hospitalization in the past year**	N/A	N/A	14.333 (1.023–200.907)	0.048

N/A = Not applicable; SC = Self-contained.
